# Antimicrobial Activity of Chrysoeriol 7 and Chochlioquinone 9, White-Backed Planthopper-Resistant Compounds, Against Rice Pathogenic Strains

**DOI:** 10.3390/biology9110382

**Published:** 2020-11-07

**Authors:** Yoon-Hee Jang, Jae-Ryoung Park, Kyung-Min Kim

**Affiliations:** Division of Plant Biosciences, School of Applied Biosciences, College of Agriculture and Life Science, Kyungpook National University, Daegu 41566, Korea; uniunnie@naver.com (Y.-H.J.); icd92@naver.com (J.-R.P.)

**Keywords:** rice, antimicrobial, white-backed planthopper, phylogenetic tree, biopesticide

## Abstract

**Simple Summary:**

This study is an important contribution to the development of biopesticide materials for controlling major pathogens in rice. Chrysoeriol 7 and cochlioquinone 9, which repel the white-backed planthopper, were extracted from rice and their antimicrobial activity was investigated. The results show they are effective in limiting the growth of pathogens of the genera *Fusarium*, *Cladosporium* and *Pythium*. This shows that they have great potential as an alternative to chemical pesticides and is also thought to be helpful in development of eco-friendly agriculture.

**Abstract:**

As environmental damage caused by chemical pesticides appears worldwide, eco-friendly agriculture is increasing, and finding eco-friendly pesticide materials has become very important. Chrysoeriol and cochlioquinone, two flavonoids, act as an antibacterial and antioxidant, and increase the resistance of rice to the white-backed planthopper (WBPH). In this experiment, chrysoeriol 7 (C7) and cochlioquinone 9 (C9) were extracted from rice inoculated with the WBPH using MeOH, and cultivars with high extraction efficiency were selected. In addition, the antimicrobial activity of C7 and C9 against various pathogens causing disease in rice was tested. The results show that C7 has antifungal activity against *Fusarium graminearum* and *Pythium graminicola*, and C9 show antifungal activity against *Cladosporium herbarum*, *Cladosporium cladosporioides*, *Gibberella zeae*, *Fusarium graminearum* and *Pythium graminicola*. When both substances were treated at a concentration of 1000 ppm, they showed high inhibition rates of 62.3% and 36.2% against *P.* *graminicola*, respectively. After that, a phylogenetic tree was created to clarify the relationship between the microorganisms whose growth was inhibited and divided into three groups. This result can contribute to the study of biopesticide materials that can control pests and pathogens.

## 1. Introduction

Rice (*Oryza sativa* L.) is the main food crop for more than 50% of the world’s population, making it one of the most important crops in the world. The largest yield of rice is obtained from Asia, where about 94% of the world’s supply is grown [1]. In a report on agricultural prospects by the OECD, FAO and other international organizations, the world’s population will reach about 9.6 billion by 2050 and demand for food will increase by more than 60% [2]. To meet the needs of the rapidly growing population, a significant increase in rice production is needed in the coming decades.

In natural environments, however, rice is easily attacked by pests, nematodes and pathogens such as viruses, bacteria and fungi [3]. These attacks cause rice diseases and result in significant yield losses worldwide, threatening rice supplies and global food security. There are more than 50 kinds of rice diseases reported in Korea [4]. When the occurrence is severe, depending on the type of disease it may lead to a direct loss in yield and quality [5]. Migratory insects also cause major problems in Asia, especially *Laodelphax striatellus* Fallen (the small brown planthopper, SBPH), *Nilaparvata lugens* (the brown planthopper, BPH) and *Sogatella furcifera* Horvath (the white-backed planthopper, WBPH), which are well known for causing serious damage in south and east Asia [6].

Since the high-yield rice variety began to replace existing Japonica-type varieties in the Green Revolution of the 1960s, the WBPH and BPH have been the major pests causing damage to rice farms in the early summer every year from the tropical climatic regions in southern China to Korea, Japan and central China [7]. Because of the damage caused by the insects, pesticides are considered an essential tool for rice production, and are indispensable for achieving agricultural productivity [8]. Nowadays, agriculture is dependent on chemical fertilizers and organic synthetic pesticides, and problems with those substances have begun to emerge around the world [9]. To avoid those problems, environmentally friendly agriculture has become an alternative. As eco-friendly cultivation areas increase, research has been actively conducted to find eco-friendly materials with pesticidal effects that do not significantly harm the environment [10].

Some plant extract compounds contain bioactive substances and show little harm to humans, so they are recognized as pesticide substitutes in environmentally friendly agriculture [11,12]. Chrysoeriol, also known as 3′-O-methylluteolin, is a 3′-O-methylated flavonoid [13]. This flavonoid has a methyl group attached to the 3 atom of the flavonoid backbone. Therefore, it is considered a flavonoid lipid molecule and is a yellow needle-shaped crystal with a melting point of 337 °C [14]. Chrysoeriol is found mostly in the leaves of *Eriodictyon glutinosum* Bentham and is also present in a wide variety of plants, vegetables [15], fruits [16] and flowers [17]. Chrysoeriol is of great interest scientifically because of its antioxidant, anti-inflammatory, antibacterial and antiviral properties. Glucoside bound to chrysoeriol is an isoflavonoid that plays an important defense against invasion of pathogens and insects [18]. Chrysoeriol has antimicrobial activity against various pathogens; particularly, it has been found to contain artemisinin derivatives, which are malaria drugs caused by *Plasmodium falciparum* [19]. Chrysoeriol glycoside isolated from *G. glandulosum* has antibacterial activity that destroys the cytoplasmic membrane by cell lysis and cytoplasmic permeability to leak the cellular components, killing the cells [20]. Chrysoeriol strongly induces the expression of heme oxygenase (HO)-1, an antioxidant enzyme, and at the same time promotes the intranuclear migration of the transcription factor *Nrf2*, thereby enhancing the antioxidant effect [21]. It also strongly inhibits the induction of nitric oxide synthase by blocking *AP-1* activation [22]. The production of antimicrobial compounds is associated with fungal-mediated insects [23,24]. 

When insects invade leaves, the fungus grows around the wounds and they produce a variety of interesting plant toxins. The fungi produce phytotoxins causing plant stress, but sometimes at the same time they also produce substances to protect plants from insect attack. A cochlioquinone is a flavonoid and an antibacterial compound of the fungus vectorized by *Clusia* spp. The WBPH induces fungi into leaves, which then produce cochlioquinone A [25]. The parasitic fungus *Cochliobolus miyabeanus* produces cochlioquinone A and B, which are yellow pigments, as small metabolites [26]. Their mixed biosynthesis occurs by introducing farnesyl units into aromatic precursors where secondary methyl groups are derived from methionine [27]. Cochlioquinone is a bioactive substance and is sometimes used as a nematode agent that competes for specific 3H ivermectin binding sites [28]. Cochlioquinone A1 (CoA1) isolated from the fungal strain *Bipolaris zeicola* is used as a potent antiangiogenic agent [29]. Kim et al. (2013) [30] used the Cheongcheong/Nagdong doubled-haploid cultivar to select the WBPH-resistant strains. Park et al. (2014) [31] performed HPLC analysis of C7 (resistive compound) identified as chrysoeriol and C9 (susceptible compound) identified as cochlioquinone 9 in rice inoculated with WBPH [30,31]. In this study, we investigated the antimicrobial activity of chrysoeriol 7 and cochlioquinone 9 against rice pathogens by extracting and separating the WBPH-resistant material from rice, and then judge their potential as eco-friendly pesticide alternatives.

## 2. Materials and Methods

### 2.1. WBPH Breeding and Plant Material

**Figure 1:**
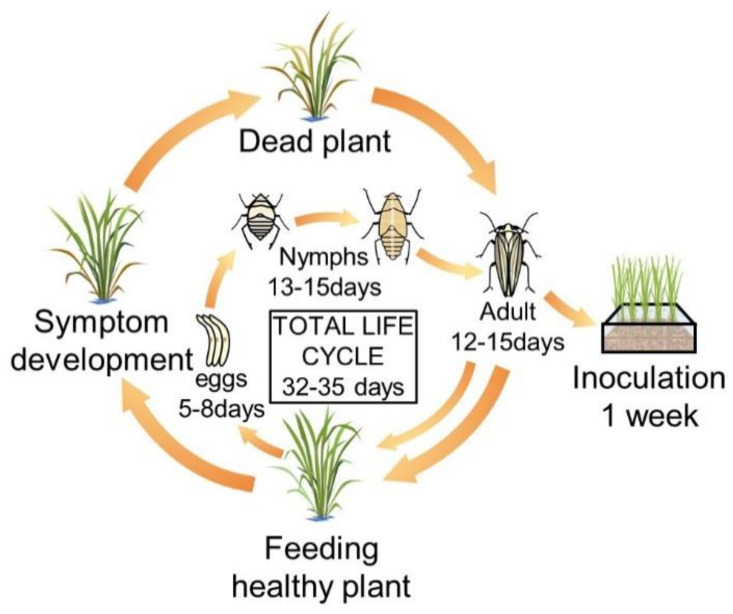
Breeding system and life cycle of the white-backed plant hopper (WBPH). In the breeding cage, healthy rice at the seedling stage was provided as food for the white-backed plant hoppers (WBPHs) every week. The WBPH hatches from eggs after 5–8 days and has a nymph period of 13 to 15 days. After that, it becomes an adult and is active for 12–15 days. At this time, if new food is provided, the adult insects move to the new food. The total life cycle of the WBPH is 32 to 35 days, and the sucked rice turns yellow and dies over time.

Fifty male and female WBPHs were obtained from the National Agricultural Science Research Institute of the Rural Development Administration (RDA). The breeding cage was made of an acrylic plate (50 × 50 × 40 cm) and a 100-μm mesh net was used on the back for ventilation and water supply. The insect nursery was maintained at a temperature of 27 °C ± 1 °C, a humidity of 60 to 70%, a luminous intensity of 2000 lux and over a 16-h cycle. Chucheong, one of the most preferred rice cultivars for WBPHs, was used for their food. Twenty to twenty-five grams of seeds were sterilized using a Spotak pesticide, then soaked in an incubator at 33 °C for 3 days and washed with clean water every morning. After sowing into a 21 cm × 14 cm × 5 cm plastic container and supplying sufficient moisture, the plants were placed in dark conditions at 28 °C for 2 to 3 days, and then 2 or 3 leaf seedlings grown in a greenhouse for 1 to 2 weeks were used as feed. The food was changed every week, which helps the next generation of WBPHs to be sustained (Figure 1). When the WBPHs were at their 2 or 3 instar, they were inoculated into the plant material, namely, Cheongcheong, Nagdong and TN1.

Cheongcheong, Nagdong and TN1 were harvested at the Kyungpook National University Experimental Field at Gunwi in Korea in 2018. Cheongcheong is a resistant cultivar, and Nagdong and TN1 are cultivars sensitive toward the WBPH. Sixty grams of seeds were sterilized with 500 μL of Spotak pesticide (HANKOOKSAMGONG, Seoul, South Korea) in 1 L of water at 33 °C for 3 days in an incubator and washed with clean water every morning. After sowing in a plastic container (21 × 14 × 5 cm) and supplying sufficient moisture, the plants were placed in dark conditions at 28 °C for 2 to 3 days and then grown in the greenhouse. The 50 WBPHs of the 2nd or 3rd instar were inoculated into Cheongcheong, Nagdong and TN1 for a week.

### 2.2. Bacterial and Fungal Pathogens

Two bacteria and 11 fungi causing rice disease were obtained from the Agricultural and Microbiology Department of the National Academy of Agricultural Science, RDA (Table 1). The freeze-dried bacteria were suspended in 5 mL of Luria–Bertani (LB) broth and cultured at 30 °C and 130 rpm for 3 days in an incubator. The freeze-dried fungi were suspended by adding 0.3–0.5 mL of sterile distilled water, and then the suspended samples were inoculated with 0.1 mL in potato dextrose agar (PDA) solid medium using a pipette and cultured at 25 °C in an incubator for one week. After culturing, the growth and contamination of the strains were confirmed, and the purely cultured strains were used for the experiment.

### 2.3. Extraction and Separation of Compounds

The leaves of Cheongcheong, Nagdong and TN1 inoculated with the WBPHs were cut and ground using a mortar with liquid nitrogen. Thirty-five grams of each leaf was used. Then, 35.0 g of plant sample and 350 mL of 70% methanol were added in a 1000 mL Erlenmeyer flask and shaken overnight in an incubator at 20 °C, 130 rpm. A filter paper (Whatman Grade 2 Qualitative Filter Paper Diameter: 15.0 cm; pore size: 8 μL) was fixed in the funnel and inserted into the Erlenmeyer flask. The sample was poured onto filter paper and this filtering was repeated twice.

The sample was washed using an equal volume of n-hexane: equal amounts of sample and n-hexane were added in a separation glass, shaken for few minutes, and then left until the layers were completely separated before collecting the bottom layer of the solution. The samples were concentrated using a rotary evaporator. The concentration was carried out under conditions of a water bath temperature of 30 °C, a cooling solution at −3 °C to 5 °C and a speed of 6–8. The samples were concentrated until the volume was about 10 mL and then concentrated more using a heating block. They were heated at 60 °C until the sample in each tube was about 0.3 mL.

One hundred and twenty-five grams of silica gel 60 were poured into a glass column (diameter 10 mm × 250 mm) using a funnel. The silica gel was first washed with 20% methanol, and then all of the concentrated sample was added in until it was soaked in silica gel. We poured the 20 mL eluent into a column and waited until the solution had fallen. The eluent gradient was 4% methanol and 20% acetone. We collected the solution in the tube until all samples had come down. We dried the collecting eluent with a heating block at 50 °C. After drying, the compound in some tubes was checked by TLC silica gel 60F_254_ plates (Merck, KGaA, Darmstadt, Germany) with a mobile phase (Chloroform:Methanol:1-Butanol:Water = 4:5:6:4) and exposed in a UV eliminator (Bio-rad, USA).

The silica matrix containing the compound was cut to separate C7 and C9. The compounds were then separated from the silica matrix using a small column and eluted by 20 mL of 4% methanol in 100% acetone to get the purified compounds. The purity of compounds was checked by TLC silica gel 60F_254_ plates.

### 2.4. LC/MS for Identification

We used LC/MS to analyze the materials, with an MSQ Plus Single Quadrupole Mass Spectrometer (Thermo Fisher Scientific, San Diego, CA Waltham, MA, USA). The infusion concentration was a 1:1000 sample dilution using 50% methanol in 0.1% formic acid and the flow rate was 50 μL/min.

### 2.5. Antimicrobial Activity Test of the Compounds 

The separated compounds were dried using a heating block, dissolved with distilled water at 3 concentrations (100 parts per millions (ppm), 500 ppm and 1000 ppm), and then 0.1 mL was applied to the medium. For the bacteria, the liquid LB medium was suspended in distilled water to have a 1.0 OD value at 600 nm using a UV spectrophotometer, and then 0.1 mL of the bacteria was dropped into the LB medium using a pipette and cultured at 30 °C in dark conditions. For the fungi, a part of the subcultured mycelium (0.5 × 0.5 cm) was transplanted to the PDA medium and cultured at 25 °C in dark conditions. After inoculation, the growth over 1 and 2 weeks was confirmed, and the diameter of the colony was measured. According to Kim et al. (2012), the following formula (1) was used for the calculation of the inhibition rate (%) [32].

(1) $$\mathrm{Inhibition}\;\mathrm{rate}\;\left(\%\right)=\;\left(1-\frac{\mathrm{Colony}\;\mathrm{diameter}\;\mathrm{in}\;\mathrm{treatment}\;\mathrm{medium}}{\mathrm{Colony}\;\mathrm{diameter}\;\mathrm{in}\;\mathrm{untreated}\;\mathrm{medium}}\right)\times 100$$

### 2.6. Statistical Analysis

All experiments were replicated at least three times, and the statistical analysis was performed using the SPSS program (IMMSPSS Statistics, version 22, IBMSPSS Statistics, version 22, Redmond, WC, USA).

### 2.7. Polymerase Chain Reaction Protocol and Sequencing

Bacterial and fungal DNA were extracted from the samples using the DNeasy Plant Mini Kit (Qiagen, Hilden, Germany; Cat. No. 69104). Polymerase chain reaction (PCR) amplifications were done in 30 μL mixtures containing 5 μL of 20–30 ng/mL template DNA, 1 μL of 10 pM of each primer, 2 μL of dNTPs, 0.3 μL Ex taq polymerase (Imclone Biotech Co., Manhattan, NY, USA), 3 μL of 10× Ex buffer and 12.7 μL of nuclease-free water (Qiagen, Cat. No. 129114). The reaction consisted of an initial denaturation for 5 min at 94 °C followed by 35 cycles for 30 s at 94 °C, annealing for 30 s at 55 °C and an extension of 72 °C for 30 s. The PCR was performed using a GeneAmp PCR System 2700 (Applied Biosystems, Waltham, MA, USA) or MyGenie96 Thermal Block (Bioneer, Daejeon, Korea). Primers were used as the universal primer 27F (5′-AGAGTTTGATCCTGGCTCAG-3′), 1492R (5′-GGTTACCTTGTTACGACTT-3′), ITS1 (5′-TCCGTAGGTGAACCTGCGG-3′) and ITS4 (5′- TCCTCCGCTTATTGATATG-C-3′). Each primer targets the bacterial 16S rRNA and the fungal ITS region. After the PCR, the products were treated by electrophoresis in 1.2% agarose gel. Amplification products were purified by a QIA quick Gel Extraction Kit (Qiagen, Cat. No. 28706). Sequencing analyses were done by SolGent Co., Ltd. (Bethesda, MD, USA). The sequencing analysis for the homology search was done using the BLAST program at NCBI (http://www.ncbi.nim.nih.gov) database.

### 2.8. Phylogenetic Analysis

The sequences were aligned using Clustal W multiple alignment. Bacterial 16S and fungal ITS sequences were entered in the MEGA X program (Philadelphia, PA, USA) for construction of the phylogenetic trees. The phylogenetic tree was constructed using the distance method, neighbor-joining. Branch support was given using 1000 bootstrap replicates.

## 3. Results

### 3.1. Extraction Efficiency by Variety

After separation of C7 and C9, 1.7 mg, 3.6 mg and 12.1 mg of C7 and 9.0 mg, 4.1 mg and 14.0 mg of C9 were obtained in Cheongcheong, Nagdong and TN1, respectively. The highest amount of both C7 and C9 were obtained from TN1, as confirmed by TLC.

### 3.2. LC/MS Data

**Figure 2:**
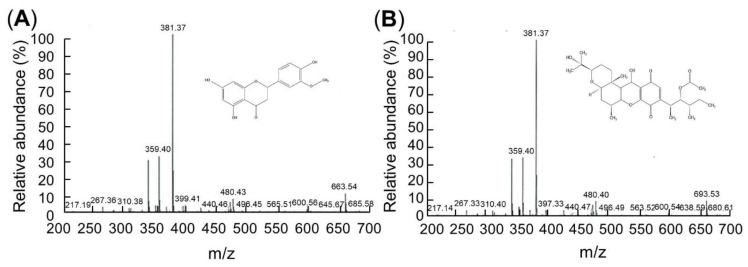
C7 and C9 LC/MS data. The flow rate was consistently 50 μL/min with a 320 °C capillary temperature and 49 V of capillary voltage. (**A**) Chrysoeriol 7 (C7). Mass: 267.36 and 381.37 *m*/*z*, 5,7-dihydroxy-2-(4-hydroxy-3-methoxyphenyl)chromen-4-one, C_16_H_12_O_6_, molecular weight = 300.26 g/mol. (**B**) Cochlioquinone 9 (C9). Mass: 381.38 and 480.40 *m*/*z*, (3R)-9-[(1S,2R,3S)-2-acetyloxy-1,3-dimethylpentyl]-1,2,3,4aβ,5,6,6a,12,12aβ,12b-decahydro-12β-hydroxy-3α-(1-hydroxy-1-methylethyl)-6aα,12bα-dimethylpyrano[3,2-a]xanthene-8,11-dione, C30H44O8, molecular weight = 532.7 g/mol.

LC/MS analyses were conducted to identify the compounds. The LC/MS chromatograms of C7 and C9 are shown in Figure 2. Positive and negative LC/MS data revealed the molecular weight to be 267.36 and 381.37 *m*/*z* in C7 and 381.38 and 480.40 *m*/*z* in C9 (Figure 2).

### 3.3. Antimicrobial Activity Test of C7 and C9

**Figure 3:**
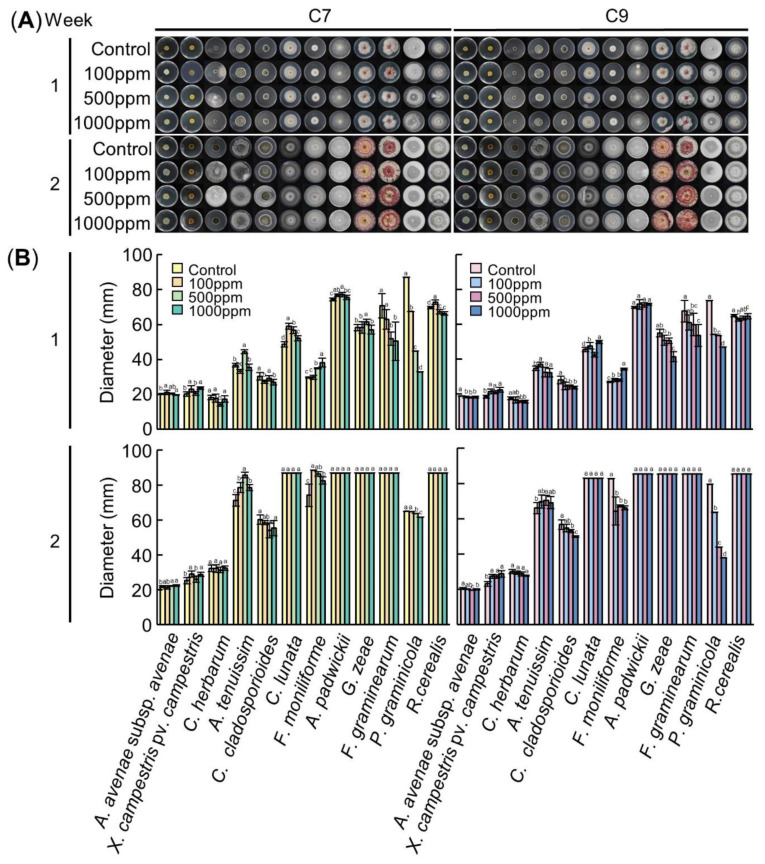
Antimicrobial activity of C7 (left panel) and C9 (right panel). (**A**)Visual growth of different bacterial and fungal strains. Bacteria were cultured in an LB medium and fungi were cultured in a PDA medium at 25 °C in a dark state. The medium was treated with C7 and C9 at concentrations of 0 ppm, 100 ppm, 500 ppm and 1000 ppm, respectively. Colonies were observed after 1 and 2 weeks of growth after inoculation. (**B**) Colony diameter according to the concentration of the compounds at 1 week and 2 weeks after inoculation. Means with the same letters are not significantly different by Duncan’s multiple range test at *p* < 0.05.

In the antimicrobial activity test of C7, the inhibition of growth of *F. graminearum* and *P. graminicola* were significantly different according to the concentration of the C7 treatment (*p* < 0.05) (Figure 3). At 1 week after inoculation, the inhibition rates of *F. graminearum* were 10.9% when treated with 100 ppm, 26.6% when treated with 500 ppm and 28.7% when treated with 1000 ppm. It was confirmed that, as the treatment concentration of C7 increased, the growth of the fungi decreased; all media were overcultured at 2 weeks. The inhibition rates of *P. graminicola* were 22.6%, 48.54% and 62.27%, showing high inhibitory effects at 1 week. However, at 2 weeks after inoculation, the inhibition rates were very low, 0.2%, 1.9% and 5.1% (Table 2 and Table 3). In the antimicrobial activity test of C9, the inhibition of growth of *C. herbarum, C. cladosporioides, G. zeae, F. graminearum* and *P. graminicola* were significantly different according to the concentration of the C9 treatment (*p* < 0.05) (Figure 3). At 1 week after inoculation, the inhibition rates of *C. herbarum* were 5.0%, 9.9% and 10.1%, respectively. At 2 weeks, the inhibition rates were 2.3%, 4.3% and 8.0%, showing a weak inhibitory effect. The inhibition rates of *C. cladosporioides* were 12.4%, 14.3% and 15.5% at 1 week and 3.6%, 6.4% and 12.0% at 2 weeks, respectively. The inhibition rates of *G. zeae* were 7.6%, 8.1% and 24.6%. With the 1000 ppm treatment, the inhibition rate increased significantly, and at 2 weeks it was overcultured. The inhibition rates of *F. graminearum* were 9.7%, 11.3% and 20.7% at 1 week, respectively, and overcultured in 2 weeks. The inhibition rates of *P. graminicola* were 26.4%, 27.3% and 36.2% at 1 week, respectively, showing a relatively high inhibitory effect. The inhibition rates at 2 weeks were 20.0%, 44.8% and 52.4%, which were higher than those at Week 1 (Table 4 and Table 5).

The growth of *F. graminearum* and *P. graminicola* was inhibited by both C7 and C9. The antifungal activity against *F. graminearum* was slightly higher in C7, and the antifungal activity against *P. graminicola* was higher in C9. Neither C7 nor C9 had an effect on the bacteria *A. avenae* subsp. *avenae* and *X. campestris* pv. *campestris.*

### 3.4. Phylogenetic Tree

**Figure 4:**
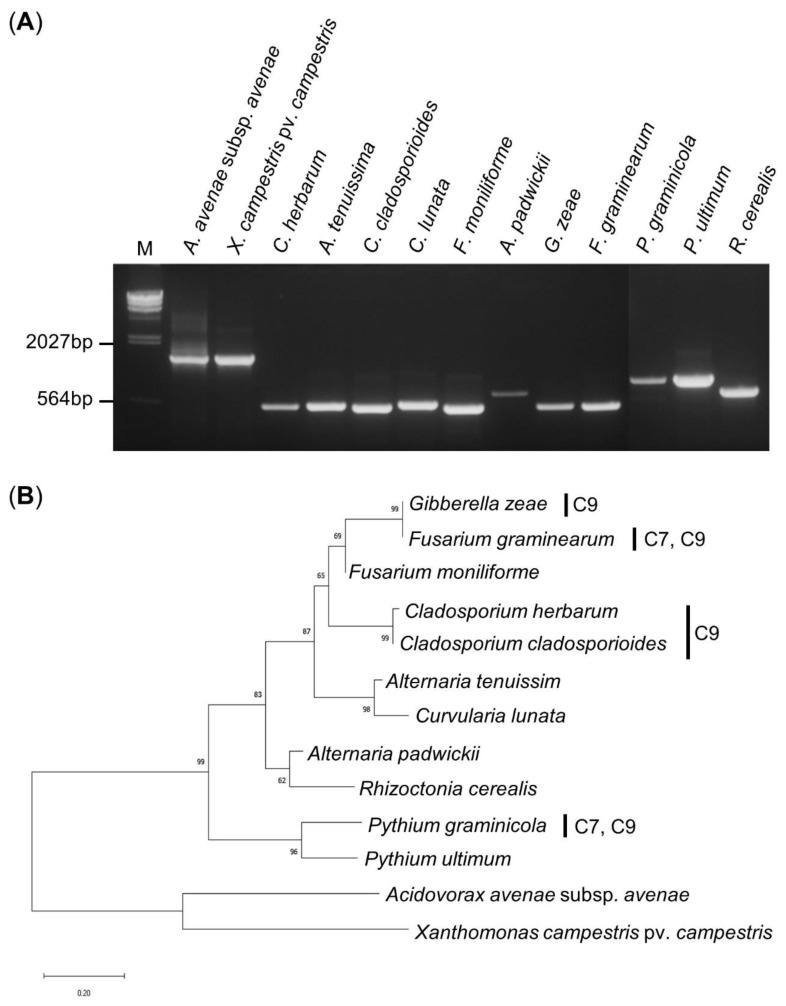
PCR amplification and phylogenetic tree. (**A**) Gel electrophoresis of the PCR products of the 16S rRNA region and ITS region using a universal primer. The PCR product was run on a 0.8% agarose gel. M: λ/*Hind* III DNA ladder. (**B**) Phylogenetic tree of the bacterial and fungal strains using the neighbor-joining method. The phylogenetic tree is based on the 16S rRNA and ITS gene sequences constructed with 1000 bootstraps in which the percentages over 50 are indicated in each node.

We obtained approximately 1500 bp 16S rRNA PCR amplification products from the 2 bacteria and 500–600 bp ITS region PCR amplification products from the 11 fungi (Figure 4a). The PCR amplified products were analyzed through the BLAST program of NCBI, and a neighbor-joining phylogenetic tree was created using the Mega X program. As expected, the bacteria and fungi were divided into two categories, phylogenetic and eukaryotic. *G. zeae* and *F. graminearum* grouped with the *Fusarium* genus with more than 99% identical nucleotide sequences. *C. herbarum* and *C. cladosporioides* showed a very high homology of 98.2% to the same *Cladosporium* genus, and the bootstrap value was very high at 99%. *G. zeae*, *F. graminearum*, *C. herbarum*, and *C. cladosporioides* could be grouped together by the *Ascomycota* phylum, but showed low homology of 64–65.8% and a bootstrap value of 65%. *P. graminicola*, which showed inhibitory effects by C7 and C9, did not belong to the *Ascomycota* phylum, and the homology was only 30.7–34.3%, showing a distant relationship (Figure 4b).

## 4. Discussion

Chemical pesticides are widely used around the world to prevent pests and pathogens from attacking crops, and they have become a major cause of environmental pollution. Chemical pesticides are very efficient and appear to act quickly, but there are problems, such as residual toxicity and increased resistance by pests. Biological pesticides are alternatives to these organic synthetic pesticides. After the Rio summit agreement to replace 20% of the world’s pesticides with biological pesticides, there is a clear movement to expand the availability of biological resources by developing new natural pesticides worldwide [33]. Among the currently discovered plant-derived biological pesticides, pyrethrin, which is eluted from pyrethrum flowers, has an insecticidal component and is mainly used to control moths and aphids [34]. Leaf extracts of *Piper betle*, *Ocimum sanctum*, *Nyctanthes arbor-tristis* and *Citrus limon* are known to inhibit the growth of pathogens of rice in vivo [35].

Chrysoeriol is an organic compound known as a 3′-O-methylated flavonoid and has a structure in which a methoxy group is attached to the c3 atom of the flavonoid backbone. It is abundantly present in many plants, fruits and medicinal herbs. Chrysoeriol is used in research on neuroprotection [36], anti-inflammatory, anticancer [37], antibacterial [38] and antioxidant [39] effects [40]. Cochlioquinone A is a major component of the bioactive pigments isolated from *Bipolaris leersia* and *D. saccharhi*. It is anti-angiogenic and has the inhibitory effects of diacylglycerol kinase and diacylglycerol acyltransferase. Cochlioquinone B is a sesquiterpene metabolite, an inhibitor of NADH ubiquinone reductase, and a phytotoxic agent that inhibits root growth of finger millet and rice at a 100 ppm concentration [26]. Cochlioquinone D is a physiologically active natural product that inhibits root growth of *Lolium multiflorum* [41]. Cochlioquinone 9 is distinguished from cochlioquinone A in that C26 (methyl group) is not bound to C14 but is bound to C15. 

In separating chrysoeriol and cochlioquinone from rice inoculated with WBPHs, it is possible to extract a large amount of material faster than when not inoculated, increasing the efficiency and reducing the cost [42]. The highest amount of C7 and C9 was extracted from TN1, a susceptible cultivar, at the seedling stage. However, the amount of material can be different depending on various factors such as the growth time, the period of inoculation and the extraction method. According to Park et al. (2014), there is a large difference in damage between resistant rice cultivars and susceptible cultivars after 25 days of inoculation [31]. In this experiment, compounds were extracted under a single condition, so further experimentation is required to determine under which conditions the C7 and C9 content of the resistant cultivars will be higher compared to other varieties. In the LC/MS analysis of the separated material in the present study, C7 showed molecular weights of 267.36 and 381.37 *m*/*z*, and C9 showed molecular weights of 381.38 and 480.40 *m*/*z*. This is consistent with the molecular weights of C7 and C9, as previously known [31]; the authors reported that the molecular weight of Chrysoeriol 7 was 267.33, 341.50, 353.42 and 381.50 *m*/*z*, and that of Cochlioquinone 9 was 381.50 and 469.42 *m*/*z*. Since the molecular weight of the material separated in this experiment almost matched, it was judged to be the same material. 

Looking at the results of the inhibition rate against the pathogens when C7 and C9 were treated with varying concentrations, when C7 was treated, the inhibition rate against *F. graminearum* at 1 week was 10.9, 26.6 and 28.7%, respectively, and the inhibition rate significantly increased when treated with 500 ppm. However, when processing 1000 ppm, it increases very little. The inhibition rate against *P. graminicola* was 22.6%, 48.54% and 62.27%, showing high inhibitory effects, and the inhibition rate was also greatly increased in proportion to the concentration of the compound. However, at 2 weeks after inoculation, the inhibition rate was very low (Table 2). When looking at the inhibition rate when C9 was treated, when 500 ppm was used in *C. herbarum* and *C. cladosporioides* at 1 week, the inhibition rate was 9.9% and 14.3%, which was the most efficient compared to the other concentration. At 2 weeks, both showed weak inhibitory effects overall. In *G. zeae*, the inhibition rate was 24.6% when treated with 1000 ppm at 1 week, which was higher than when 500 ppm was used. After 1 week, the inhibition rate of *F. graminearum* and *P. graminicola* increased significantly to 20.7% and 36.2%, respectively, when 1000 ppm was used, and the inhibition rate of *P. graminicola* further increased after 2 weeks (Table 3). C7 has an inhibitory effect against two pathogens, *F. graminearum* and *P. graminicola*, and C9 has an inhibitory effect against a wider range of fungi, including *F. graminearum* and *P. graminicola*, but the inhibitory effect was higher in C7. The most efficient control concentration was different, depending on the type of pathogen when the compounds were treated, but the most efficient treatment concentration for simultaneous control of several fungal pathogens was 500 ppm.

The bacterial 16S rRNA and the fungal ITS region were used to investigate the genetic similarity between microbes whose growth was inhibited by C7 and C9 through the phylogenetic tree. Most of the 16S rRNA sequences are quite conserved, although high sequence diversity appears in some sections. While there is little diversity between homogeneous species, diversity appears between other species, so it is used to identify prokaryotes [43]. The length of the ITS region of the fungus is not long, so it can be easily analyzed. It has been widely used for systematic analysis because it can be used even among nearby taxa due to its rapid mutation rate [44]. As a result of systematic analysis, microorganisms were largely divided into three groups. *G. zeae* and *F. graminearum* cause scab in rice and produce fungal toxins that affect rice safety and are toxic to the immune system and digestive system [45]. The outbreak of mycotoxins caused by contamination of *F. graminearum* has been reported steadily around the world, and it is estimated that 25% of the world’s grain production, including rice, is contaminated with mycotoxins [46]. In Korea, scab disease caused by *F. graminearum* occurred widely in the southern regions during the harvesting season in 1963, resulting in a reduction of yield of 40–60% in some regions and 80–100% in severe regions [47]. *C. herbarum* and *C. cladosporioides* are species in the genus *Cladosporium*, which causes spotting symptoms on crops, causes allergies to livestock and promotes asthma. In addition, each pathogen causes false blast and ear blight, which directly damages the ear of rice, not only reducing the quantity but also the quality of the harvest [48]. *P. graminicola* is a plant pathogen that infects grains such as barley, wheat, rice and beans. In rice, it causes blight [49]. Damping-off caused by *P. graminicola* is a disease that affects the early growth of the seedling, infringing on the branch of the hypocotyl and causing the seedling to fall. In the end, it falls and dies. When a lot of damping-off occurs, the seedlings are insufficient, which causes great damage to the planting [50]. However, we found no antimicrobial effect of C7 and C9 on *P. ultimum*, which has 83.7% homology with *P. graminicola*, so this needs to be confirmed. 

Research using microorganisms, plant extracts and natural enemies is being carried out to develop biological pesticides to replace chemical pesticides and develop eco-friendly agriculture. However, the production of plant extracts is small; thus, the amount that can be used for pesticides in areas with high demands is extremely limited. The extracts can be effective and could be used as biological pesticides and antibacterial agents if a mass extraction method and an automatic analysis system for substances are established in the future.

## 5. Conclusions

In this study, in order to overcome the environmental damage caused by chemical pesticides, we tried to find eco-friendly biopesticide material that can control pests and pathogens. Chrysoeriol 7 and cochlioquinone 9, which are insect-resistant compounds, were extracted from rice and their antimicrobial activity against major pathogens causing disease in rice was examined. The results showed that chrysoeriol 7 had antifungal activity against *Fusarium graminearum* and *Pythium graminicola* and cochlioquinone 9 had antifungal activity against *Cladosporium herbarum, Cladosporium cladosporioides, Gibberella zeae, Fusarium graminearum* and *Pythium graminicola*. These results indicate that they are effective in limiting the growth of some pathogens and show their potential as alternatives to harmful chemical pesticides. These findings can also contribute to the basic research of eco-friendly materials to use as pesticides in rice farming.

## Figures and Tables

**Table 1:** List of bacteria and fungi obtained from the Rural Development Administration (RDA).

Pathogens	Scientific Name	Disease Name	KACC No.	Media	Temperature (°C)
Bacteria	*Acidovorax avenae* subsp. *avenae*	Bacterial Stripes	16205	LB	28
	*Xanthomonas campestris* pv. *campestris*	Bacterial Leaf Blight	10377	LB	30
Fungi	*Cladosporium herbarum*	False Rice Blast	42599	PDA	26
	*Alternaria tenuissima*	Ear Blight	40968	PDA	30
	*Cladosporium cladosporioides*	Ear Blight	40934	PDA	25
	*Curvularia lunata*	Ear Blight	40392	PDA	24
	*Fusarium moniliforme*	Ear Blight	40386	PDA	25
	*Alternaria padwickii*	Ear Blight	43247	PDA	25
	*Gibberella zeae*	Scab	46523	PDA	25
	*Fusarium graminearum*	Scab	41040	PDA	25
	*Pythium graminicola*	Damping-off	40155	PDA	25
	*Pythium ultimum*	Damping-off	40705	PDA	25
	*Rhizoctonia cerealis*	Sheath Eyespot	40153	PDA	25

**Table 2:** Inhibition rate (%) of chrysoeriol 7 against various pathogen species.

Species	Inhibition Rate (%)					
	1 Week			2 Weeks		
	100 ppm	500 ppm	1000 ppm	100 ppm	500 ppm	1000 ppm
*A. avenae* subsp. *avenae*	−4.5	−1.5	2.6	2.2	−1.4	−3.2
*X. campestris* pv. *campestris*	−13.5	−1.4	−16.3	−15.0	−3.5	−14.5
*C. herbarum*	2.2	21.9	4.7	0.2	2.8	−0.6
*A. tenuissima*	9.6	−21.1	3.5	−10.3	−20.5	−10.3
*C. cladosporioides*	9.9	3.3	10.9	2.9	10.2	8.2
*C. lunata*	−21.1	−16.6	−7.1	0.0	0.0	0.0
*F. moniliforme*	−0.6	−18.1	−29.0	−19.4	−16.6	−11.4
*A. padwickii*	−3.2	−3.8	−1.6	0.0	0.0	0.0
*G. zeae*	−0.2	−5.7	2.1	0.0	0.0	0.0
*F. graminearum*	10.9	26.6	28.7	0.0	0.0	0.0
*P. graminicola*	22.6	48.5	62.3	0.2	1.9	5.1
*R. cerealis*	−4.4	3.4	4.8	0.0	0.0	0.0

**Table 3:** Diameter of radial growth of various pathogen species against chrysoeriol 7.

Species	Diameter (mm)									
	1 Week					2 Weeks				
	Control	100 ppm	500 ppm	1000 ppm	*p* Value	Control	100 ppm	500 ppm	1000 ppm	*p* Value
*A. avenae* subsp. *avenae*	20.1 ± 0.4b ^z^	21.1 ± 1.3a	20.4 ± 0.4ab	19.6 ± 0.3b	0.019 *	21.7 ± 0.5b	21.3 ± 1.0ab	22.0 ± 0.3a	22.4 ± 0.5a	0.025*
*X. campestris* pv. *campestris*	20.3 ± 1.3b	23.0 ± 1.8a	20.5 ± 1.2b	23.5 ± 0.8a	<0.001 **	25.1 ± 1.8b	28.9 ± 1.6a	26.0 ± 1.9b	28.8 ± 1.2a	0.001 **
*C. herbarum*	18.2 ± 1.3a	17.8 ± 2.3a	14.2 ± 1.0b	17.3 ± 1.7a	0.002 **	32.1 ± 1.8	32.1 ± 2.2	31.3 ± 1.6	32.4 ± 1.3	0.722
*A. tenuissima*	36.7 ± 1.0b	33.2 ± 1.1bc	44.4 ± 1.1a	35.4 ± 1.8b	<0.001 **	71.2 ± 3.3c	78.6 ± 3.0b	85.8 ± 1.5a	78.6 ± 1.8b	<0.001 **
*C. cladosporioides*	30.2 ± 2.2a	27.2 ± 1.0b	29.2 ± 1.5a	26.9 ± 1.6b	0.006 **	60.1 ± 2.7a	58.4 ± 1.1ab	54.0 ± 4.3bc	55.2 ± 4.4c	0.020 *
*C. lunata*	48.6 ± 1.5d	58.9 ± 1.7a	56.7 ± 2.0b	52.1 ± 1.5c	<0.001 **	87.0 ± 0.0	87.0 ± 0.0	87.0 ± 0.0	87.0 ± 0.0	1.000
*F. moniliforme*	29.5 ± 0.3c	29.7 ± 1.2c	34.9 ± 0.3b	38.1 ± 2.5a	<0.001 **	74.1 ± 6.3c	88.5 ± 0.0a	86.4 ± 1.2ab	82.6 ± 2.0b	<0.001 **
*A. padwickii*	74.4 ± 0.8c	76.7 ± 0.7ab	77.1 ± 1.3a	75.6 ± 1.3bc	0.001 **	87.0 ± 0.0	87.0 ± 0.0	87.0 ± 0.0	87.0 ± 0.0	1.000
*G. zeae*	58.2 ± 1.8b	58.3 ± 3.4b	61.5 ± 1.4a	57.0 ± 2.5b	0.024 *	87.0 ± 0.0	87.0 ± 0.0	87.0 ± 0.0	87.0 ± 0.0	1.000
*F. graminearum*	70.6 ± 7.0a	62.9 ± 5.7a	51.8 ± 3.9b	50.4 ± 11.1b	<0.001 **	87.0 ± 0.0	87.0 ± 0.0	87.0 ± 0.0	87.0 ± 0.0	1.000
*P. graminicola*	87.0 ± 0.8a	67.4 ± 0.2b	44.8 ± 0.5c	32.8 ± 0.4d	<0.001 **	64.9 ± 0.7a	64.8 ± 0.4a	63.7 ± 0.4b	61.6 ± 0.6c	<0.001 **
*R. cerealis*	69.7 ± 0.7b	72.7 ± 1.2a	67.3 ± 1.2c	66.3 ± 1.1c	<0.001 **	87.0 ± 0.0	87.0 ± 0.0	87.0 ± 0.0	87.0 ± 0.0	1.000

^z^ The data are presented as the mean ± standard deviation. Means with the same letters are not significantly different by Duncan’s multiple range test at *p* < 0.05. * significant at the 0.05 level. ** significant at the 0.01 level.

**Table 4:** Inhibition rate (%) of cochlioquinone 9 against various pathogen species.

Species	Inhibition Rate (%)					
	1 Week			2 Weeks		
	100 ppm	500 ppm	1000 ppm	100 ppm	500 ppm	1000 ppm
*A. avenae* subsp. *avenae*	3.6	4.8	3.9	1.7	4.5	2.1
*X. campestris* pv. *campestris*	−16.8	−14.3	−21.5	−18.5	−17.6	−24.5
*C. herbarum*	5.0	9.9	10.1	2.3	4.3	8.0
*A. tenuissima*	−6.4	6.3	7.1	−5.6	−6.7	−4.7
*C. cladosporioides*	12.4	14.3	15.5	3.6	6.4	12.0
*C. lunata*	−4.9	6.8	−9.5	0.0	0.0	0.0
*F. moniliforme*	−3.9	−3.6	−27.1	22.2	18.7	20.0
*A. padwickii*	−2.5	−1.9	−2.6	0.0	0.0	0.0
*G. zeae*	7.5	8.1	24.6	0.0	0.0	0.0
*F. graminearum*	9.7	11.3	20.7	0.0	0.0	0.0
*P. graminicola*	26.4	27.3	36.2	20.0	44.8	52.4
*R. cerealis*	3.4	2.1	0.4	0.0	0.0	0.0

**Table 5:** Diameter of radial growth of various pathogen species against chrysoeriol 9.

Species	Diameter (mm)									
	1 Week					2 Weeks				
	Control	100 ppm	500 ppm	1000 ppm	*p* Value	Control	100 ppm	500 ppm	1000 ppm	*p* Value
*A. avenae* subsp. *avenae*	19.0 ± 0.2a ^z^	18.3 ± 0.4b	18.1 ± 0.3b	18.3 ± 0.6b	0.006 **	20.5 ± 0.5a	20.2 ± 0.3ab	19.6 ± 0.2c	20.1 ± 0.3b	0.025 *
*X. campestris* pv. *campestris*	18.5 ± 0.9b	21.6 ± 1.3a	21.1 ± 1.0a	22.5 ± 1.4a	<0.001 **	23.2 ± 1.3b	27.5 ± 1.1a	27.2 ± 1.0a	28.8 ± 1.8a	0.001 **
*C. herbarum*	17.6 ± 0.8	16.7 ± 1.7	15.8 ± 0.5	15.8 ± 0.8	0.107	30.1 ± 1.1a	29.4 ± 0.9ab	28.8 ± 1.2bc	27.7 ± 0.3	0.722
*A. tenuissima*	34.8 ± 1.3b	37.0 ± 1.4a	32.6 ± 2.7a	32.3 ± 2.5a	<0.001 **	66.0 ± 3.1b	69.7 ± 3.7ab	70.5 ± 2.7a	69.2 ± 3.3ab	<0.001 **
*C. cladosporioides*	28.2 ± 2.1a	24.7 ± 2.2b	24.1 ± 1.2b	23.8 ± 0.8b	0.001 **	56.8 ± 2.7a	54.7 ± 2.1ab	53.1 ± 0.9b	49.9 ± 0.4c	0.020 *
*C. lunata*	45.5 ± 1.3d	47.8 ± 1.8b	42.4 ± 1.2c	49.9 ± 1.0a	<0.001 **	83.0 ± 0.0	83.0 ± 0.0	83.0 ± 0.0	83.0 ± 0.0	1.000
*F. moniliforme*	27.0 ± 0.3c	28.1 ± 0.9b	28.0 ± 0.7b	34.3 ± 0.6a	<0.001 **	82.8 ± 0.0a	64.4 ± 7.9b	67.3 ± 0.6b	66.2 ± 1.2b	<0.001 **
*A. padwickii*	69.7 ± 0.6	71.4 ± 2.8	71.0 ± 1.2	71.5 ± 0.5	0.185	85.3 ± 0.0	85.3 ± 0.0	85.3 ± 0.0	85.3 ± 0.0	1.000
*G. zeae*	55.0 ± 2.2ab	50.9 ± 2.9b	50.6 ± 1.6b	41.5 ± 2.9c	<0.001 **	85.3 ± 0.0	85.3 ± 0.0	85.3 ± 0.0	85.3 ± 0.0	1.000
*F. graminearum*	67.5 ± 6.2a	61.0 ± 4.4ab	59.9 ± 6.4bc	53.5 ± 6.3c	0.005 **	85.3 ± 0.0	85.3 ± 0.0	85.3 ± 0.0	85.3 ± 0.0	1.000
*P. graminicola*	73.7 ± 0.3a	54.2 ± 0.6b	53.6 ± 0.4c	47.0 ± 0.5d	<0.001 **	79.6 ± 0.4a	63.7 ± 0.6b	44.0 ± 0.5c	37.9 ± 0.5d	<0.001 **
*R. cerealis*	64.9 ± 0.7a	62.7 ± 0.9b	63.6 ± 1.2ab	64.6 ± 1.5a	0.010 **	85.3 ± 0.0	85.3 ± 0.0	85.3 ± 0.0	85.3 ± 0.0	1.000

^z^ The data are presented as the mean ± standard deviation. Means with the same letters are not significantly different by Duncan’s multiple range test at *p* < 0.05. * significant at the 0.05 level. ** significant at the 0.01 level.
